# Persisting Differences or Adaptation to German Fertility Patterns? First and Second Birth Behavior of the 1.5 and Second Generation Turkish Migrants in Germany

**DOI:** 10.1007/s11577-015-0331-8

**Published:** 2015-09-21

**Authors:** Sandra Krapf, Katharina Wolf

**Affiliations:** 1 Institut für Soziologie und Sozialpsychologie (ISS), Universität zu Köln, Greinstraße 2, 50939 Cologne, Germany; 2 Department of Demography & Max Planck Institute for Demographic Research, Rijksuniversiteit Groningen, Konrad-Zuse-Str. 1, 18057 Rostock, Germany; 3 Population Research Centre, Faculty of Spatial Sciences, University of Groningen, Landleven 1, 9747 AD Groningen, The Netherlands

**Keywords:** Migrants’ descendants, Fertility, Second generation, 1.5 generation, Turkish migrants, Adaptation, Socialization, Germany, Nachkommen von Migranten, Fertilität, Zweite Generation, Generation 1,5, Türkische Migranten, Adaption, Sozialisation, Deutschland

## Abstract

In this study, we use data of the German Mikrozensus to explore first and second birth behavior of migrants’ descendants. Whereas prior waves of the Mikrozensus only included respondents’ citizenship, in the survey years 2005 and 2009 also parental citizenship has been surveyed. This allows us to identify respondents’ migrant backgrounds, even if they have German citizenship. We distinguish those who migrated as children (1.5 generation) from those who were born to Turkish parents in Germany (second generation migrants). We compare both migrant generations to German non-migrants. Using discrete-time hazard models, our results show that 1.5 generation migrants have the highest probability of having a first and second birth, while German non-migrants have the lowest birth probabilities. The second generation lies in-between. This pattern also persists after taking the educational attainment of respondents into consideration. However, there seems to be an adaptation of highly educated second generation Turkish migrants to non-migrant Germans: we find no significant differences in the probability of having a first birth in the two groups. For second births, we do not find this pattern which might be related to the young age structure in the sample of second generation migrants.

## Abstract (de)

In der vorliegenden Studie analysieren wir auf Basis des Mikrozensus das Erst- und Zweitgeburtsverhalten der Nachkommen türkischer Migrantinnen in Deutschland. In früheren Wellen des Mikrozensus wurde zur Identifikation des Migrationshintergrundes lediglich die Staatsbürgerschaft erfragt. Die Mikrozensuswellen 2005 und 2009 geben nun erstmals Auskunft darüber, welche Staatsbürgerschaft die Eltern der Befragten haben, sodass sich Nachfahren von Migranten auch identifizieren lassen nachdem sie die deutsche Staatsbürgerschaft angenommen haben. In der Analyse unterscheiden wir zwischen türkischen Migrantinnen, die im Kindesalter migrierten (1,5te Generation), und jenen, die von türkischen Eltern in Deutschland geboren wurden (zweite Migrantengeneration). Beide Gruppen werden Frauen in Deutschland ohne Migrationshintergrund gegenübergestellt. Ergebnisse eines diskreten Hazardmodells zeigen, dass die 1,5te Migrantengeneration die höchste und Nicht-Migrantinnen die niedrigste Wahrscheinlichkeit aufweisen, ein erstes oder zweites Kind zu bekommen. Die Wahrscheinlichkeit einer Geburt in der zweiten Migrantengeneration liegt zwischen denen der anderen beiden Gruppen. Auch nach Kontrolle für das Bildungsniveau der Frau bleiben diese Muster bestehen. Allerdings scheint eine Anpassung hochgebildeter Migrantinnen der zweiten Generation an das Verhalten von Nicht-Migranten stattzufinden: Zwischen beiden Gruppen sind unter den hochgebildeten Frauen keine signifikanten Unterschiede in der Erstgeburtswahrscheinlichkeit zu finden. Beim Übergang zur zweiten Geburt ist dieser Effekt eines hohen Bildungsstands weniger stark ausgeprägt.

## Introduction

On average Germany has experienced positive net migration in the last few decades, and the stock of foreign people living in the country has been growing since the mid-twentieth century (Destatis 2013, 2014). The majority of international migrants arrived from Mediterranean countries (e.g., from Turkey, Italy, and Greece) in the context of labor migration in the 1960s and early 1970s, and for family reunion thereafter. Today, migrants with Turkish roots form the largest immigrant group originating from a single country, representing 3.6 % of the total population in Germany (Destatis 2012). The special situation of international migrants moving from one cultural background to the other provides an insight into integration processes and social change (Kalter 2003). Migrant behavior is often examined by focusing on the question of whether migrants adapt to behavioral patterns in the receiving society. In this vein, labor market integration (Granato and Kalter 2001; Konietzka and Seibert 2003; Seibert and Solga 2005), educational adaptation (Fick 2011; Groh-Samberg et al. 2012; Segeritz et al. 2010), and patterns of life satisfaction among migrants (Safi 2010; Siegert 2013; Zapf and Brachtl 1984) have been under study. One aspect that has been less explored is the demographic adaptation of migrants in Germany. This is of specific interest for migrants from high fertility countries, such as Turkey. A large body of international research has investigated the childbearing behavior of migrants, showing that the timing of migration, the duration of stay, the reasons to migrate and a person’s labor force participation affect migrant fertility (Andersson 2004; Andersson and Scott 2005, 2007; Cygan-Rehm 2011; Mayer and Riphahn 2000; Milewski 2007; Mussino and Strozza 2012; Toulemon 2004; Wolf 2014). These studies focus on the first migrant generation, i.e. those who migrated as adults.

In order to better understand the integration processes across migrant generations, we analyze fertility patterns of Turkish migrants’ descendants. In the 2000s, children of labor migrants reached ages of 30 years or older. Although they have not yet completed their reproductive phase, their fertility behavior in their thirties is already indicative for overall fertility. This study compares non-migrant Germans and descendants of Turkish migrants. We distinguish between the second generation, i.e. those who have migrant parents but who were born in the country of destination, and the so-called 1.5 generation, i.e. those who migrated as children. Our central research questions are: How do first and second birth patterns of non-migrant Germans, 1.5, and second generation Turkish migrants differ? Are fertility differences between migrants and non-migrants caused by differences in the socio-economic composition of the groups?

Analyzing those who migrated as children separately is promising in two respects. On the one hand, selectivity issues or disruption arguments are less relevant for the 1.5 generation migrants because they did not take the decision to migrate themselves. While the first generation, who migrated as adults, might consciously time their decision to migrate and to start a family, for the 1.5 generation the migration and fertility transitions can be assumed to be independent of one another. Their fertility should not be distorted by migration timing, as is the case for migrants who arrived during their childbearing years (Toulemon 2004; Wolf 2014). Accordingly, selection into migration is less relevant for the 1.5 generation and biases are avoided (Adsera et al. 2012). On the other hand, contrasting second and 1.5 generation migrants allows us to single out the effect of childhood socialization, as this is the main distinction of these two groups. The 1.5 generation was partly exposed to family values in the country of origin whereas the second generation experienced their entire childhood in the country of destination. Therefore, variations in fertility behaviour between the two groups are likely to be the result of different socialization environments.

Our analyses are based on the German Mikrozensus. The large sample size allows us to study the descendants of Turkish migrants as a single migrant group. We use two Mikrozensus waves from the years 2005 and 2009. In other survey years, migration information was limited to citizenship and year of migration, which made it impossible to identify second generation migrants with German citizenship. The extended question program in 2005 and 2009 allows us to identify these second generation migrants. Using the own-children method, we generate the age at childbirth. We compare the transition to first and second birth among women of the two migrant groups to non-migrant western Germans, i.e. respondents who were born in Germany and whose parents were non-migrants. By employing event history techniques, we control for standard socio-demographic characteristics, such as education. Although it would have been interesting to also analyze third birth behavior, only a very selective group is at risk of having a third birth as particularly the second but also the 1.5 generation are rather young (see Table 5 in the appendix).

## Theoretical consideration

Especially those migrants who decide to stay are of great importance for the demographic development of a country because the group of stayers affect population development. This leads to the question of how far integration progresses and what the determinants are. A first attempt to present a theoretical framework was made by representatives of the Chicago School, who developed an approach to explain assimilation processes in the US (Gordon 1964; Park and Burgess 1921). The *classical assimilation theory* describes the decline of an ethnic or racial distinction and the cultural and social differences that express it (Alba and Nee 1997). Assimilation was expected to be an inevitable, gradual process which increases over immigrant generations (Alba and Nee 1997; Zhou 1997). However, the theory received a lot of criticism. It was argued that receiving societies are not homogenous and that migrants might adapt to specific groups rather than to mainstream society, resulting in *segmented assimilation* (Portes and Zhou 1993; Rumbaut 1994). Moreover, it was criticized that both classical assimilation and segmented assimilation theory do not offer explicit mechanisms to explain assimilation processes, but they merely describe empirical outcomes (Esser 2004, 2008). Others observed that the concept of assimilation in general implies a dominance of the majority society (Bade and Bommes 2004). Thus, in Europe since the 1980s, researchers prefer the normatively more neutral concept of integration to the term assimilation (Aumüller 2009, p. 34). Social integration can be conceptualized as a “process of inclusion and acceptance of migrants in the core institutions, relations and statuses of the receiving society” (Heckmann 2006, p. 18). The processes can refer to first generation immigrants as well as to their children and grandchildren (ibid.: p. 17).

The fertility patterns of migrants can serve as an indicator of integration into the society in the country of destination (Coleman 1994). Fertility decisions are influenced by both cultural and structural conditions (Lesthaeghe and Surkyn 1988; Letablier et al. 2009; Rindfuss and Brewster 1996). The two mechanisms can differ between countries, which might result in diverse fertility patterns across countries. If migrants follow their home country’s predominant fertility behaviour, this can lead to fertility differentials between migrants and non-migrants in the country of destination. A number of theoretical arguments have been suggested to explain the fertility behavior of first generation migrants, such as the socialization, adaptation, disruption, and selection hypotheses (Kulu 2005; Kulu and González-Ferrer 2013; Lindstrom and Giorguli Saucedo 2007). However, there is less research on the fertility behavior of migrants’ descendants. We argue that comparing second and 1.5 generation is promising because neither of the groups has taken the decision to migrate on their own. Therefore, disruption effects do not play a role in their fertility patterns. While for the first generation it was argued that Turkish migrants are a selective group with rather low socio-economic background, this should be of minor relevance for the descendants of migrants. It has been shown that they also differ systematically in their socio-economic situation from non-migrants in the country of destination. However, the effect of the parents’ socio-economic background on children’s characteristics should be similar for both the 1.5 and second generation, and comparing the two groups should not lead to distortions due to selectivity. In the following, we discuss how socialization, adaptation and composition effects might explain differences in fertility behavior among non-migrants, second, and 1.5 generation migrants.

### Childhood socialization

Family values and gender role attitudes differ across countries (Nauck and Klaus 2007). Based on socialization theory, researchers expect that these social roles and values are transmitted to each social group member via socialization (Goode 1964). In the classic formulation of the theory, socialization is described as a process that takes place largely within the family and during childhood (Parsons 1955). Family-related norms and values are also transmitted during childhood within the family (Putney and Bengtson 2002). In line with this, it has been shown that mothers pass on their gender role attitudes (Moen et al. 1997), and their childbearing preferences (Barber 2000) to their daughters.

Concerning international immigrants, it is argued that the home country’s norms and values regarding fertility preferences persist even after migration. Empirical evidence has shown that those who migrated from high fertility origin countries have considerably higher fertility than non-migrants in the low fertility destination countries (Alders 2000 for the Netherlands; Andersson 2004 for Sweden; Kahn 1988 for the US). However, fertility norms and values are also transmitted via the first generation to their children. In line with this, it was found that first generation migrants transmit their higher child number ideals and lower age norms concerning the first child to their children (Nauck 2001; Nauck et al. 1997). Also for female migrants in the Netherlands, studies have indicated that children reproduce their parents’ preferences for an early entry into motherhood (De Valk 2006; De Valk and Liefbroer 2007). These attitudes are mirrored in fertility patterns: the second generation of Turkish migrants shows higher first birth rates than do the majority populations in several European countries (Milewski 2011). Moreover, a study of Germany indicates that second generation migrants are on average younger at first birth than non-migrant (western) Germans, but are older than first generation migrants (Milewski 2010a).

Socialization arguments explain not only why migrants and their descendants show different fertility behaviour than non-migrants. They also provide a framework to explain why migrant generations are distinct. Based on the fact that the 1.5 generation was born in Turkey and the second generation migrants were born in Germany, the two groups have partly different socialization experiences. Both groups are influenced by the Turkish community and family in the country of destination. But those migrating as children were partly socialized in the country of origin, i.e. they were exposed to their home countries’ norms to a larger extent than those born in the host country. By contrast, the second generation experienced socialization entirely in the receiving society. They maintained social contacts with both peers of Turkish origin and non-migrant Germans during childhood and were thus exposed to German family norms to some extent. Also their parents had been living longer in the receiving society and might have adapted to the host country norms themselves. Because of their different socialization experiences during childhood, we expect that 1.5 generation Turkish migrants are more likely to have a child than non-migrants and that the second generation takes on an intermediate position between the two groups (hypothesis 1).

### Adaptation

While socialization arguments are usually employed to explain behavioural differences between migrant generations and non-migrants, adaptation arguments help us to understand why fertility patterns converge. Adaptation consists of two different mechanisms that are interrelated and affect one another (Frank and Heuveline 2005; Kulu 2005; Rumbaut and Weeks 1986). On the one hand, the economic conditions in the country of destination affect childbearing. From a neo-classical micro-economic perspective, fertility decisions are the product of direct costs and opportunity costs of children (Becker 1991; Hotz et al. 1997; Mincer 1963). Moving to a country with better job perspectives for women and higher living costs increases the costs of childrearing for migrants from less developed areas. Accordingly, they adapt their fertility behavior toward lower fertility and later birth transitions. In line with this, studies in Sweden have shown that women participating in the labour market have largely the same fertility patterns—independent of migrant background (Andersson and Scott 2005, 2007). On the other hand, fertility is determined by norms and values concerning the ideal family size and the timing of parenthood. According to Hoffman and Hoffman’s (1973) “Values of Children”-approach, the “value of children refers to the functions they serve or the needs they fulfill for parents” (ibid.: 46 f.). Empirically, it has been shown that the value parents attach to children differs systematically across countries (Nauck 2007; Nauck and Klaus 2007). In a similar vein, the Second Demographic Transition-approach links the cultural change seen in many European countries over the last decades, marked by secular individualization trends, with decreasing fertility levels (Lesthaeghe 1995; Sobotka 2008; Van De Kaa 1994). Non-western migrants are exposed to these individualistic norms and values after migrating to European countries. They might adapt to the lower child number ideals and preferences for later entry into parenthood prevalent in the country of destination.

Initially, the concept of adaptation was used to explain adjustment processes of first generation immigrants in the short-term. The degree of adaptation was assumed to increase the longer a migrant resides in the receiving society (Hervitz 1985; Kahn 1988; Lindstrom and Giorguli Saucedo 2002; Singley and Landale 1998; Stephen and Bean 1992). But adaptation theory can also be translated to immigrants’ children. For their entire adult life, both the 1.5 and the second migrant generation are exposed to the normative and economic conditions in the country of destination. They might thus experience cultural adaptation via social contacts with the majority population, affecting their childbearing preferences. Migrants’ descendants are subject to the receiving society’s institutions and labour markets, which impacts the opportunity structure and thus childbearing. In line with this, it has been shown that across Europe second generation migrants reported higher ideal ages at parenthood than the first generation (Holland and De Valk 2013).

The adaptation of norms and values somehow contradicts the socialization theory in its original sense, where fertility preferences are assumed to be based on childhood socialization and stay constant over the life course. Nevertheless, socialization can be seen as a lifelong process, as individuals change their preferences and attitudes even after the beginning of adulthood (Mortimer and Simmons 1978; Settersten Jr. 2002). With a focus on the adult life, the adaptation theory states that the relevance of the conditions in the receiving society exceed the influence of the fertility preferences absorbed during childhood socialization. Both second and 1.5 generation migrants were exposed to German norms and conditions their entire adult lives, thus we have no reason to expect differences between 1.5 and second generation Turkish migrants (hypothesis 2).

### Compositional effects

Turkish migrants have a different socio-economic, cultural and demographic background than non-migrant Germans, and these aspects are relevant for childbearing decisions. Therefore, the composition of migrant groups could be responsible for fertility differentials. In addition to cultural factors, such as religion, language, and family orientation, the differences between migrants and non-migrants in the country of destination lie particularly in the socio-economic sphere. One indicator to approximate the socio-economic status of a person is his or her level of educational attainment. From a micro-economic perspective, higher educational levels are related to higher opportunity costs and lead to lower fertility (Schultz 1969). This negative effect is also reflected in elevated postponement of first births among highly educated and career-oriented women (Gustafsson 2001). Concerning higher order births, the relationship seems to be more complex. For some western European countries, it has been shown that education was positively related to second and/or third birth risks (Kreyenfeld and Konietzka 2008; Lappegård and Rønsen 2005; Tesching 2012).

For migrants and their descendants, it has been found that on average second generation migrants attend school longer than first generation migrants (Dustmann et al. 2012), while the educational gradient among non-migrant Germans, the second, and the 1.5 generation persists (Fick 2011). Following the composition hypothesis these educational differences would account for differences in fertility patterns of migrants and non-migrants. Based on such compositional effects, there are no reasons to expect that differences in birth risks among non-migrant Germans, 1.5 and second generation Turkish migrants persist after accounting for the effect of education (hypothesis 3).

## Turkish migrants and their descendants in Germany

Immigration from Turkey to Germany was induced by large labor shortages in Germany after World War II. To acquire foreign workers, the German government initiated agreements with several Mediterranean countries: Italy (1955), Spain and Greece (1960), Morocco (1963), Portugal (1964), Tunisia (1965) and former Yugoslavia (1968). The contract on coordinated labor migration from Turkey to Germany was signed in 1961. Most labor migrants from Turkey came from agrarian regions and had vocational qualifications for jobs in craft industries. Thus they had higher qualifications than the average Turkish population, but lower education than the average non-migrant German (Treichler 1998). Once in Germany, labor migrants filled mostly unskilled and semi-skilled jobs in industry (Seifert 1997). After the oil price shock and the resulting recession in 1973, the recruitment agreements were terminated. In the following phase, the only option to emigrate legally from Turkey to Germany was to rely on the right of family reunification or to ask for political asylum. For family reunification, an immigrant living in Germany was allowed to bring a foreign spouse and children up to age 15 to the country. As a result, the size of the foreign population in Germany increased and its composition changed (Heckmann 2003). Before 1973, immigrants were primarily workers aged between 20 and 40, most of them men. Later, more and more women and children migrated for family reunion (Münz et al. 1999).

Today, Turkish migrants and their descendants represent 3.6 % of the total German population (Destatis 2012). About half of them belong to the first immigrant generation and migrated themselves, the second generation makes up the other half (Destatis 2012). Turkish migrants and their descendants primarily live in western Germany, particularly in urban areas (Haug et al. 2009). In regard to religion, Turkish migrants form quite a homogeneous group, as more than 80 % are Muslim (Haug et al. 2009). On average, first generation Turkish migrants show lower educational degrees than non-migrant Germans (Müller and Stanat 2006; Segeritz et al. 2010). In addition, vocational qualification is low. Among Turkish women of the first migrant generation, fewer than 10 % have a vocational degree that is recognized in Germany. This is partly due to the limited transferability of degrees, because roughly 4 % of Turkish first generation women have a vocational degree that is not recognized in Germany. However, these levels also reflect the fact that obtaining vocational qualification was less common in their regions of origin in the past, particularly for women. A large share of first generation Turkish women, about 85 %, never obtained any vocational degree (Stichs 2008). This low level of qualification also affects migrants’ position in the labor market. It was found that immigrants in Germany have easier access to blue-collar jobs than to white-collar jobs (Seifert 1996). The picture is different for the second migrant generation. Because they grew up and obtained their educational degrees in Germany, their qualifications do not need to be transferred to the German system. On average, they obtain higher educational degrees and vocational education more often than do first generation migrants. However, compared to non-migrant Germans, their educational and vocational status remains lower (Müller and Stanat 2006; Segeritz et al. 2010; Stichs 2008). The 1.5 generation lies in between, in that they obtained a higher educational status than their parents, but are on average less educated compared to the second generation (Fick 2011; Segeritz et al. 2010; Seibert 2008).^1^ Altogether, socio-economic differences among Turkish migrants of the 1.5 and second generation and German non-migrants persist, and may possibly explain fertility distinctions in these groups.

In addition to the socio-economic status, family norms and values in the country of origin play an important role for migrant fertility. In the case of Turkish migrants, their religious and cultural factors differ considerably from those prevalent in Germany. In Turkey, social change has been dramatic since the beginning of the twentieth century, resulting in large disparities across social groups, who experience this change at different paces (Nauck 2002). There is no homogeneous development in Turkish society, as a situation of continuity and change has led to a hybridity of western and indigenous values (Kavas und Thornton 2013). In Turkey there is a strong belief in the concept of marriage, which is shown by undiminished marriage rates and the still extensive influence of parents on partner selection and marriage (Nauck und Klaus 2008). Intergenerational ties are still strong and it is expected that children help their parents when they are old (Nauck 2002). Nevertheless it has been reported that the value of children has been shifted from a focus on the economic advantage of children, e.g. in form of (material and non-material) help for parents, toward children’s psychological value (Kagitcibasi und Ataca 2005). The psychological value of children lies in the emotional rewards expected from having children, which is often related to a lower number of children (Nauck und Klaus 2007). In line with this, Turkish society has seen a sharp fertility decline since the beginning in the mid-twentieth century. The average total fertility rate (TFR) fell from 6.62 in the period 1950–1955 to 2.16—close to replacement level—in 2005–2010 (United Nations 2012). Despite the strong reduction in period fertility, only 10 % of women age 35 were childless in the year 2003 (Yavuz 2008), and a survey among university students in Ankara has shown that the social acceptance of childlessness is still low (Çopur und Koropeckyj-Cox 2010).

Compared to a TFR in Germany of approximately 1.4 since the 1970s, fertility in Turkey is still high. But within Turkey, there are large differences across ethnic groups. Particularly Kurdish women show much higher rates of having a higher order birth than do women of other ethnicities (Yavuz 2008). Moreover, there is also a strong educational gradient: women with high education have lower fertility than those with less education (Yavuz 2008; Nauck 2002). In addition, fertility behavior differs by region. Women living in urban regions experience the transition to first, second, and third childbirth less often and later in their life course compared to women living in rural areas (Eryurt and Koç 2012), and fertility rates are still considerbaly higher in the east than in the west of the country (Nauck und Klaus 2008). The heterogeneity of fertility patterns in Turkey across regions and ethnic groups makes it difficult to evaluate socialization arguments. Unfortunately, our data contain no information on the region of origin nor on the social environment of a person.

## Data and methods

### Data

Our analyses are based on pooled cross-sectional data from the German Mikrozensus of the years 2005 and 2009. In these two years, the household survey’s obligatory question program was extended. Prior to that, migrants could be identified only on the basis of citizenship and place of birth, meaning that descendants of migrants who were born in Germany and who had German citizenship could not be identified. In the 2005 and 2009 questionnaires a number of items refer to parents’ migration status, which allows us to distinguish the second generation even if respondents have German citizenship.

The Mikrozensus is a one-percent sample of all German households, covering standard socio-demographic characteristics such as age, citizenship, region of residence, educational attainment, etc. The scientific use file contains a 70 % subsample of the Mikrozensus data. While other studies often pool migrants from different countries of origin, the large sample size of the Mikrozensus enables Turkish migrants to be differentiated from other migrant groups. Moreover, in comparison with other surveys, nonresponse is of minor relevance in the Mikrozensus because participation is not voluntary; respondents are required by law to submit information. Unfortunately, the detailed information collected in the survey refers only to the household members, not to persons who do not live in the household. Therefore, no complete fertility histories are provided. Instead, the number of children born per woman needs to be estimated via the number of co-residing children. We reconstructed women’s fertility histories by means of the so-called “own-children method”, based on the year of birth of the mother and the year of birth of each child living in the household. This procedure might underestimate the true number of children a person has, especially in cases where a child has already left the parental home. It has been shown for respondents living in western Germany that the numbers of children calculated on basis of the “own-children method” are largely consistent with the reported numbers of biological children up to a maternal age of 40 in the Mikrozensus 2008 (Krapf and Kreyenfeld 2015). This limits our analysis to children co-residing with women in the age range 18 to 40 years, i.e. childbirths that take place beyond age 40 are not considered. Another limitation of the data is related to the fact that respondents’ characteristics refer only to the time of interview, which means we cannot account for time-varying covariates.

The vast majority of people of foreign origin migrated to western Germany and continue to live there (Destatis 2012; Münz et al. 1999). As fertility patterns differ between eastern and western Germans (Huinink et al. 2012), we compare those with Turkish background to non-migrants living in western Germany, excluding respondents living in eastern Germany from our analyses. Moreover, we do not consider respondents who are not of a Turkish or German background. This leaves us with a sample of 85,570 respondents, the vast majority of which are non-migrant Germans (82,651) and two smaller samples of 1.5 generation migrants (1130) and second generation migrants (1789).

### Methods

In a first descriptive step, we use Kaplan-Meier survival curves to compare the fertility behavior of respondents of migrant origin and non-migrant Germans. In the multivariate analyses, we run discrete-time hazard models. For the transition to first birth, the process time is the age of woman. The information on the age at first birth is generated based on the difference between the mother’s birth year and the year of birth of the oldest child in the household. For the transition to second birth, the duration since birth of the first child denotes the process time. It is calculated using the difference in the birth year of the oldest and the second-oldest child living in the household. As the yearly birth information does not allow us to distinguish between twin births and two consecutive births in a time frame smaller than 12 months, we excluded the respective respondents from the analysis of second births. Because our time scale is discrete, and assuming that the underlying latent time variable was continuous, we specified the hazard rate as complementary log-log (cloglog) function (Allison 1982). The data are organized in person-year format, with each person potentially contributing one entry per year. Cases are censored in the year a woman gives birth or when a respondent has not yet had a first (second) birth at time of the interview.

To identify whether education has a different effect on fertility patterns among non-migrant Germans and the descendants of migrants, we also interact the level of education with migrant status (two-way interaction). Moreover, we run three-way interactions in order to account for the fertility intensities by age according to educational group. It has been shown that women with lower educational levels have their highest first birth risks in their mid-twenties, while those with higher education levels enter motherhood at later ages on average (Tesching 2012). In order to examine whether these age patterns differ according to migrant background, we interact the level of education, migrant status and the age of first birth. It has to be noted that for this model we reduced the number of age groups to three (18–25, 26–32, 33–40 years). This was necessary because of the small sample size, especially for respondents of Turkish origin in the high education group. Due to sample size issues we also refrain from running the three-way-interaction for second births.

### Explanatory variables

In the multivariate analyses, the key variable is the migration background of a woman. We define three groups: non-migrants include respondents who were born in Germany and whose parents have or had exclusively German citizenship. Second generation migrants were born in Germany, but their parents have or had Turkish citizenship.^2^ The third group comprises generation 1.5, who were born in Turkey, migrated to Germany as a child and who have or had Turkish citizenship. Respondents are categorized as 1.5 generation if they migrated before age 15. It would have been interesting to investigate the behavior of those with one Turkish and one German parent, but this group was too small for any meaningful analysis and was therefore excluded from the sample. Also those who had a parent with other than Turkish or German citizenship were not considered in the analyses.

Both the woman’s birth cohort and age at birth are relevant determinants of fertility decisions. We define three cohorts: born in 1965–1972, 1973–1979 or 1980–1991. The age at birth was generated and grouped into four categories (18–24 years, 25–29 years, 30–34 years and 35–40 years). In our sample, the migrant groups differ regarding their age structure. Respondents of the second generation are younger than 1.5 generation migrants and non-migrant Germans. For both the 1.5 and the second generation, we find that the majority of observations in our sample for the transition to a first birth belong to the birth cohort 1970–1979. While more than one third of the second generation belong to the youngest cohort (born 1980–1991), this is the case for only about 14 % among generation 1.5 (see Table 3 in the appendix). The reason for this is simple: Turkish women immigrating after 1973 came primarily in the context of a family reunion (Münz et al. 1999). They arrived with their children under age 16 years, who belong to the generation 1.5. Second generation migrants were generally born after that time, and in the two Mikrozensus waves of 2005 and 2009 they had not yet reached the age of 40 years (see Table 3 in the appendix). As only a small number of second generation migrants in the data were born before 1965, we restrict the sample to those born afterwards. This leaves us with respondents born between 1965 and 1991.

In the analyses of the transition to second birth, the focus is on the age of first child at time of second birth. It has been shown that non-migrants have their first child later than those of migrant origin. In order to evaluate differences in birth timing between Turkish migrant’s descendants and non-migrant Germans, we also control for the age at first birth.

Another variable of interest is education. As mentioned before, the variables in the Mikrozensus are available only for the time of interview. Assuming that the women’s school education was completed in early adulthood, we create three categories for education: lower secondary or no school degree (low), secondary education (medium) and higher secondary education (high). The number of respondents who were enrolled in school at the time of the interview was very small. As this group had not yet gained a degree, we categorized them into the lower secondary school group. The descriptive statistics show that in our sample, non-migrant Germans have the highest level of education compared to 1.5 and second generation migrants. This is the case for both the sample for the first birth and the sample for the second birth analyses (see Tables 3 and 4 in the appendix). While only a small share of respondents of the 1.5 generation had high education (first birth sample: 17.7 %, second birth sample: 6.2 %), this share has increased for the second generation (first birth sample: 29.8 %, second birth sample: 9.2 %).

## Results

### Descriptive Results

**Fig. 1:**
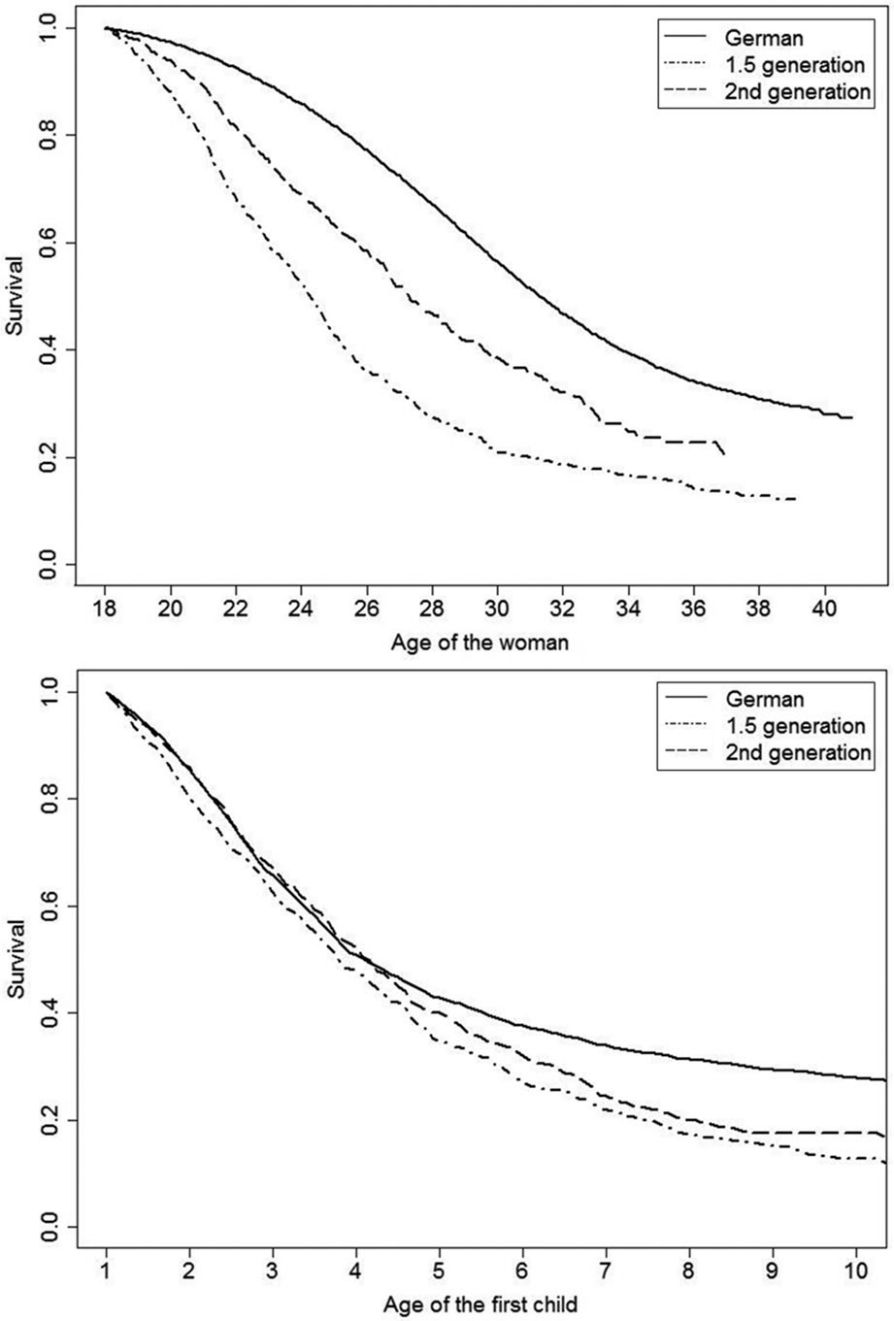
Survival curves. Non-migrant Germans, 1.5 and second generation migrants. Female respondents of birth cohorts 1965–1991. (Source: Mikrozensus 2005 and 2009, unweighted. Authors’ own illustration)

As a first step, we compare first and second births based on survival curves. However, using yearly time information results in an overestimation of the Kaplan-Meier survival estimates. In order to reduce this overestimation, we imputed a random birth month. Figure 1 describes the pattern of the transition to first and second births on basis of the pooled Mikrozensus data for the years 2005 and 2009. The first panel shows the estimated Kaplan-Meier survival curves for first births. For Germans, the median age at first birth was reached at 31.3 years. For 1.5 generation migrants the median age was 24.3, while for second generation migrants it was 27.3 years. This shows that the 1.5 generation migrants in Germany had their first childbirth earlier compared to non-migrants, while second generation migrants lie in between. In our sample, second generation migrants are still quite young; only few of them had reached ages above 38 at time of interview. The level of childlessness at age 37 is highest among non-migrants, lower for the second generation and lowest for the 1.5 generation. In order to investigate whether the different cohort composition of the three groups under study is responsible for the different fertility patterns, we compared the survival curves by five year birth cohorts (born 1965–1969, 1970–1974, 1975–1979, 1980 − 984). Although the number of exposure and occurrences was small, this sensitivity check revealed that within each cohort, the second generation remained on the intermediate position found in Fig. 1.^3^

The second panel of Fig. 1 illustrates the transition to second birth. Here, the process time of interest is the duration since first birth. For all three migrant status groups, the likelihood of having a second child is highest one to four years after the first child was born. The curves for the three groups follow a similar pattern for these first four years, with the 1.5 having the second birth a bit faster than the other two groups. For Germans, the process slows down after four years, while for Turkish descendants it continues. On average, this divergence of the survival curves after four years since first birth suggests longer birth spacing intervals for Turkish descendants compared to non-migrant Germans.

In sum, women with Turkish origin seem to start childbearing earlier and space their subsequent births further apart than do non-migrant Germans. Also, for the transition to second births, sensitivity checks for each birth cohort supported our results.^4^

### Multivariate Analyses

This section presents the results of the discrete-time hazard models on the transition to first and second births (see Tables 1 and 2). The covariates for the first and second birth models are introduced to the regression models stepwise, hence the results are presented as average marginal effects (AME), which are preferable to odds ratios when interpreting results of nested models (Best und Wolf 2012; Mood 2010). For our categorical independent variable, the AME indicates by how much the predicted probability of having a child changes on average for the respective variable value.

Model 1a shows a hump-shaped effect of age: The annual probability to have a first birth is low for respondents under age 25, rises for those between 25 and 34 years, and diminishes again for those in the age group 35 to 40 years. For birth cohort, we find a negative effect: Women born earlier have a higher annual probability of having a first birth than those born in younger birth cohorts. This indicates that there is an on-going postponement of first births. Concerning the migration background of respondents, we defined second generation migrants as a reference category in order to not only show the difference between those with Turkish origin and non-migrants, but also to evaluate whether there are significant differences between the two migrant generations. Our results indicate that the annual probability of non-migrant Germans is lower (AME = − 0.037), while that of the 1.5 generation migrants is higher (AME = 0.032) compared to respondents of the second generation (reference). In Model 1b, we additionally control for respondents’ education. We find a negative educational gradient: the higher the school education, the lower the annual probability of having a first birth. The effect of migration status is slightly reduced compared to model 1 but remains significant. This reveals that fertility differentials of non-migrants, second and 1.5 generation migrants are not fully explained by the educational composition of the three groups.

**Fig. 2:**
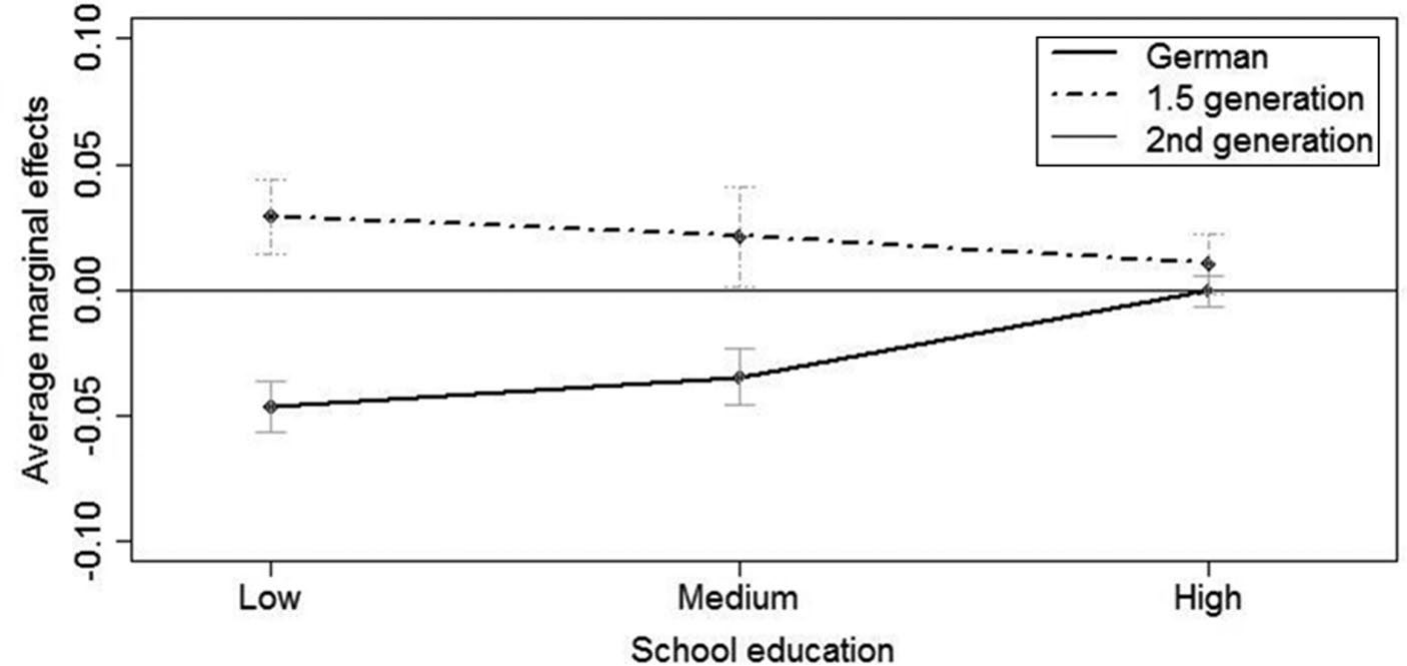
Interaction migration background and education. Transition to first birth. Discrete-time hazard model (Model 2). Average marginal effects. (Source: Mikrozensus 2005 and 2009, unweighted. Authors’ own illustrations. Notes: Controlled for mother’s age, cohort)

In order to identify whether the effect of education on first births differs across migrant generations, in Model 2 we include a two-way interaction effect of migrant background and educational attainment. Figure 2 displays the AME graphically with the second generation as reference group. The corresponding numbers are shown in Table 6 in the appendix. It reveals that Germans have the lowest annual probability of having a first birth, followed by second generation Turkish migrants, while respondents of the 1.5 generation have the highest annual probability of having a first child. However, the difference between the three migrant status groups converges over school education. While the difference is largest among women in the low education group, it is less pronounced for women with medium education and diminishes for those with high education. Among highly educated women the three migrant status groups do not differ regarding their annual probabilities of having a first birth.

**Table 1:** Determinants of the transition to first births. Discrete-time hazard model. Average marginal effects. (Source: Mikrozensus 2005 and 2009, unweighted. Authors’ own calculations)

	Model 1a	Model 1b
*Age*		
18–24	− 0.038***	− 0.041***
25–29	Ref.	Ref.
30–34	0.009***	0.010***
35–40	− 0.024***	− 0.025***
*Cohort*		
1965–1972	0.007***	0.003***
1973–1979	Ref.	Ref.
1980–1991	− 0.013***	− 0.013***
*Migration background*		
German	− 0.037***	− 0.025***
1.5 generation Turkish	0.032***	0.022***
2nd generation Turkish	Ref.	Ref.
*School education*		
Low		0.017***
Medium		Ref.
High		− 0.024***
Person years	732,371	732,371
Number of events	31,784	31,784

*p < = 0.10; **p < = 0.05; ***p < = 0.01

**Fig. 3:**
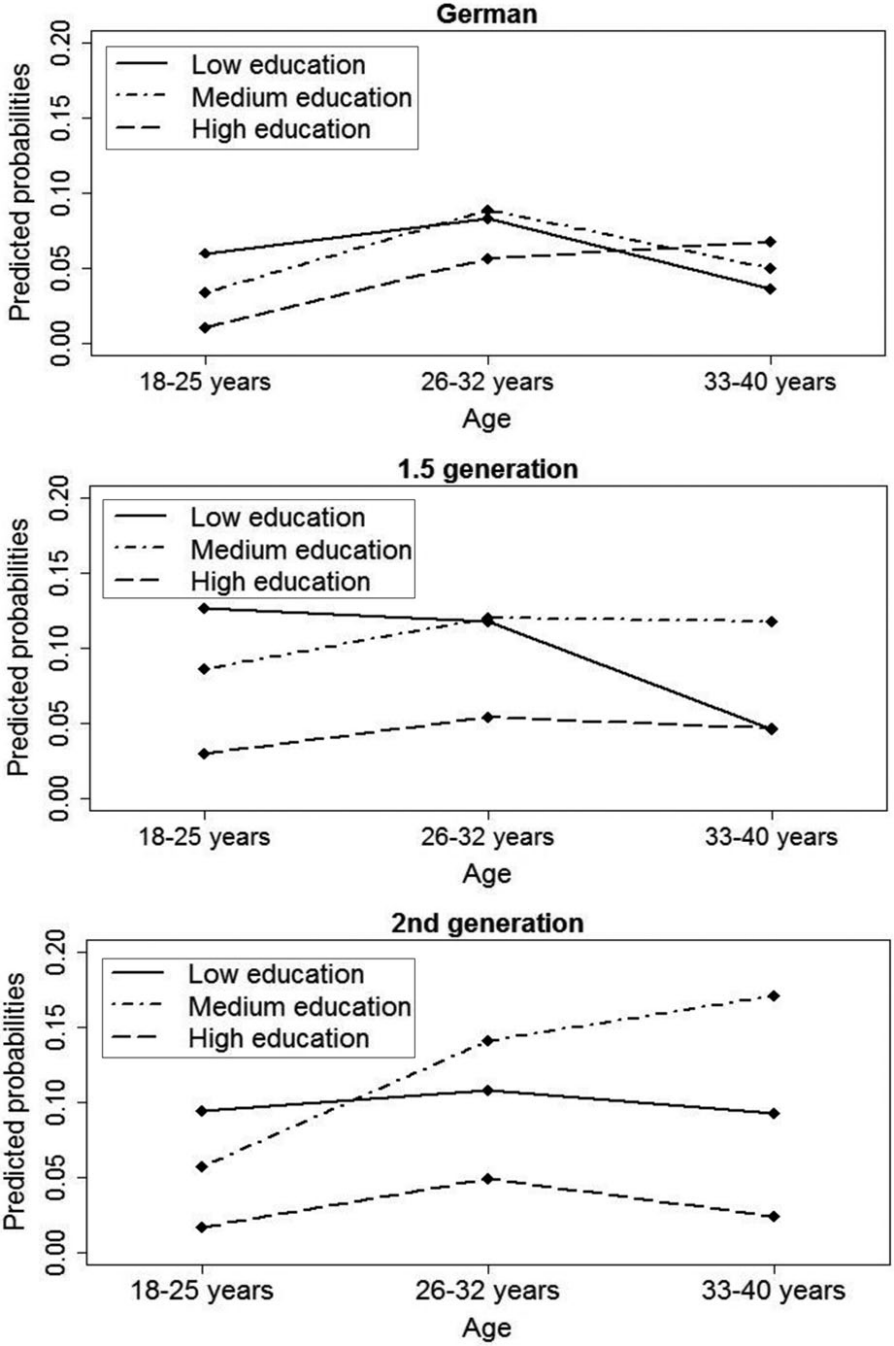
Three-way interaction of migration status, education and age. Transition to first birth. Discrete-time hazard model. Predicted probabilities. (Source: Mikrozensus 2005 and 2009, unweighted. Authors’ own illustrations)

Other studies have shown that each education group follows different fertility patterns over age (Tesching 2012). In order to identify how these patterns vary for migrants and non-migrants, we estimated three-way interaction models of education, migrant status, and age. Due to the small sample size, the occurrence of first birth in some categories was rare and therefore we reduced the number of age groups from four to three (cf. Table 7 in the appendix). Figure 3 shows the results of the three-way interaction by migrant status (Table 8 in the appendix presents these numbers). We display predicted probabilities because we are interested in the absolute probabilities of having a first child for all our age and educational groups. This allows us to identify age patterns for women with low, medium or high education in each migration status group. Average marginal effects, by contrast, would show the average effect of age and education on the probability of having a first child in comparison to one specific reference group (e.g., second generation migrants). This would not reveal the age patterns for childbearing in each migrant group—which was the focus of our three-way interaction model. The first panel of Fig. 3 presents the pattern of non-migrant Germans. For highly educated German women the probability of having a first birth rises with increasing age. They postpone first birth and have the highest annual fertility probability in the age group 33–40 years. They are also more likely to have a first birth in this age category compared to other education groups. By contrast, first childbirth among non-migrants with low or medium education is highest in the medium age group of 26–32 years. The pattern for descendants of Turkish migrants differs markedly from that of German non-migrants: Panel 2 of Fig. 3 shows that the probability of first birth of the 1.5 generation with high education remains low across all age groups. Women with a low educational level seem to show higher annual probabilities for first birth in the younger age groups. By contrast, women in the medium education category are more likely to give birth with increasing age. For the second generation (Panel 3 in Fig. 3), we find yet another pattern. The annual probability of having a first child among highly educated women is again lowest compared to other education groups and peaks at ages 26–32 years. Women with lower levels of education show nearly constant birth probabilities over age, while women with a medium level of education have highest probabilities of first birth in the oldest age group.

To summarize, the finding for highly educated non-migrant Germans indicate a postponement of first childbirth into higher ages, as was also found in previous works on western countries (Blossfeld and Huinink 1991; Ní Bhrolcháin and Beaujouan 2012). For Turkish descendants, we see no postponement of first births among the highly educated, but their fertility level remains low across all age groups compared to those with lower education. For both the 1.5 and second generation migrants, the pattern for women with a medium level of education seems to resemble that of highly educated Germans, showing an increasing probability of having a first birth over age. This effect is more pronounced among Turkish descendants of the second generation. For the interpretation, however, we have to keep in mind that the results, especially for highly educated women in the highest age group, refer to a small number of women in our sample (see also Table 7 in the appendix). This is related to two aspects: First, a smaller number of Turkish origin women have higher education. Second, Turkish migrants’ descendants are still very young and are only now reaching the ages of 35 and above.

**Fig. 4:**
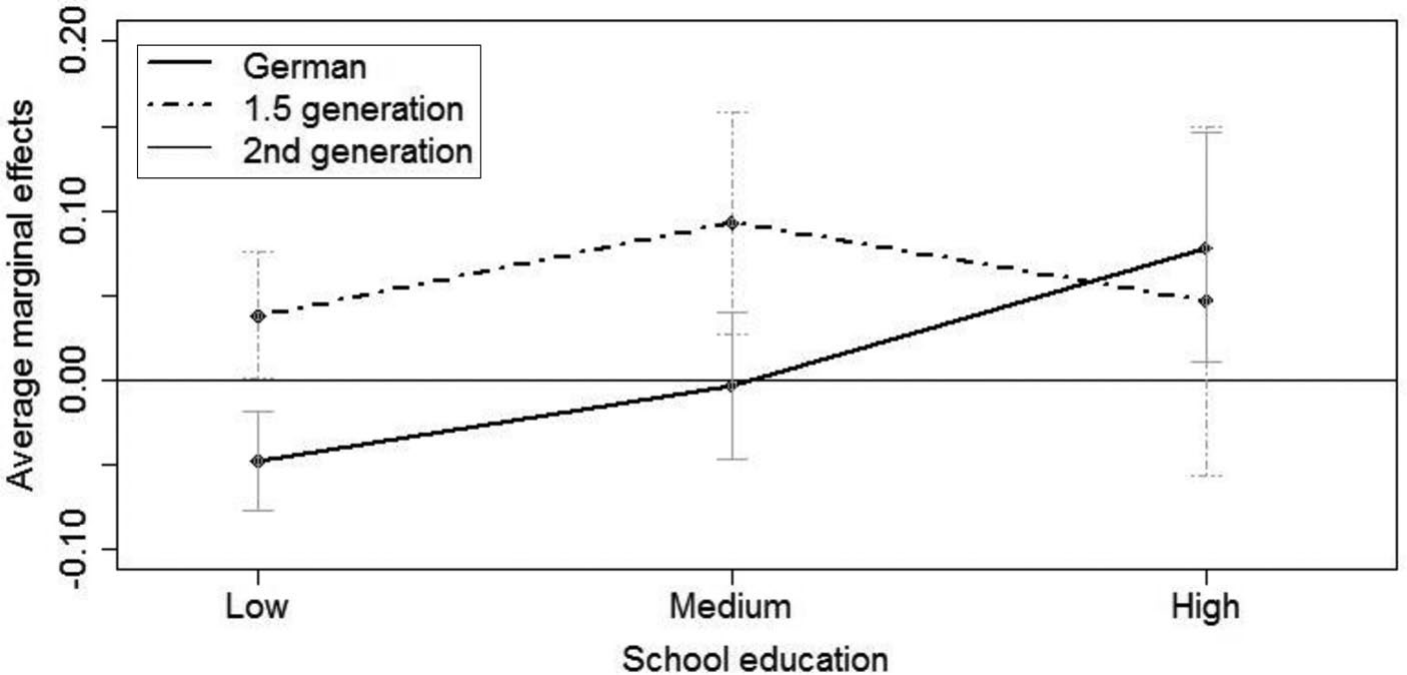
Interaction model of migration background and education. Transition to second birth. Average marginal effects (Model 5). (Source: Mikrozensus 2005 and 2009, unweighted. Authors’ own calculations. Notes: Controlled for duration since first birth, mother’s age at first birth, mother’s birth cohort)

Table 2 presents the results of the discrete-time hazard model on the transition to second birth. In these models the process time is the duration since first birth. It is shown that the probability of second birth is highest two to four years after first birth. Before and after that, the probability is lower. We also control for maternal age at first birth. In line with other studies (e.g. Kreyenfeld 2002), we find that women who had their first child after age 30 have lower annual probabilities of having a second child compared to those who were younger. Model 4a indicates a higher probability of second birth for the 1.5 generation (AME = 0.054) and no significant difference for non-migrant Germans compared to respondents of the second generation (reference). In Model 4b, we control for the educational attainment of respondents. Our results imply that for second births, women with low and medium levels of education show similar annual birth probabilities. By contrast, highly educated mothers have significantly higher annual birth probabilities compared to those with medium education (AME = 0.042). In order to identify whether this pattern is different for respondents with Turkish origin and non-migrant Germans, we specify an interaction effect (Model 5), which is graphically displayed in Fig. 4 (numbers are shown in Table 9 in the appendix). Again, second generation migrants mark the reference group. The graph indicates that the positive effect of high education is found only for Germans, whose annual probability of having a second child is significantly higher compared to second generation migrants with high education. One caveat of our analysis is that, although we were using the largest survey dataset available in Germany, we still ran into sample size problems. These sample size restrictions limit our ability to analyze interaction effects in greater detail. This is also the reason why we have refrained from running the three-way-interaction models for second births.

**Table 2:** Determinants of the transition to second births. Discrete-time hazard model. Average marginal effects. (Source: Mikrozensus 2005 and 2009, unweighted. Authors’ own calculations)

	Model 4a	Model 4b
*Years since first birth*		
1-<2	− 0.242***	− 0.242***
2-<4	Ref.	Ref.
4-<7	− 0.079***	− 0.078***
7-<10	− 0.232***	− 0.232***
10+	− 0.264***	− 0.264***
*Mother’s age at first childbirth*		
18–24	− 0.002	0.005
25–29	Ref.	Ref.
30–34	− 0.030***	− 0.036***
35–40	− 0.071***	− 0.080***
*Birth cohort*		
1965–1972	− 0.002	0.001
1973–1979	Ref.	Ref.
1980–1991	− 0.046***	− 0.044***
*Migration background*		
German	− 0.019	− 0.024*
1.5 generation Turkish	0.054***	0.057***
2nd generation Turkish	Ref.	Ref.
*School education*		
Low		− 0.006
Medium		Ref.
High		0.042***
Person years	70,768	70,768
Number of events	17,613	17,613

*p < = 0.10; **p < = 0.05; ***p < = 0.01

## Discussion

Germany has been one of the major receiving countries for migrants in Europe. The labour migrants who arrived in the 1960s and early 1970s partially remained in Germany, formed their families and had children. Therefore, the study of integration processes is increasingly reaching a stage where also the behaviour of 1.5 and second generation migrants can be analysed. Focusing on descendants of Turkish migrants, who are the largest migrant group from a single country of origin in Germany, we were interested if differences persist or fertility patterns adapt. This is an interesting endeavour because social integration of migrants is a topic of public interest. Beyond that, the socialization and adaptation processes allow us to learn something about the interplay of normative and institutional determinants of social change (Kalter 2003).

Based on data of the German Mikrozensus, this study focused on fertility patterns of the 1.5 and second generation Turkish migrants compared to non-migrant western Germans. Our results show that the 1.5 generation, who migrated as children, have the highest annual probability of having a first child; Germans have the lowest probability, while the second generation lie in between these two groups.

The comparison of the second and 1.5 generations allows us to disentangle adaptation and socialization effects. According to adaptation theory, the destination country’s childbearing values and its opportunity structure influence migrants’ fertility behaviour. Because both groups, the 1.5 generation as well as the second generation, have spent their entire adult life in Germany, they should adapt to the low fertility patterns of non-migrant Germans to the same extent. Alternatively, socialization theory expects that the migrant generations differ because the generation 1.5 had been partly socialized in Turkey, while the second generation spent its entire childhood in Germany. Our analyses show that 1.5 generation migrants differ markedly from the German pattern, while the fertility behaviour of the second generation is more similar to that of non-migrants. This is in line with the socialization hypothesis. The fertility differentials between the two migrant generations indicate that family values learnt through childhood socialisation are of great importance and play a role in later fertility behaviour of migrants’ descendants. This finding does not necessarily contradict adaptation arguments, but it seems that socialization effects are more relevant here.

We find adaptation tendencies of fertility particularly among highly educated women. For those with lower education, the annual probability of having a first birth varies strongly between non-migrants, 1.5 and second generation migrants, while the difference diminished slightly for those with medium education. Among highly educated women, annual first birth probabilities do not differ across the three migrant status groups. It seems that differences between German non-migrants and Turkish descendants of the 1.5 and second generation are partly caused by women’s educational background. Our findings indicate that high education has an equalizing effect, i.e. that the effect of a migrant background vanishes for women with high education. Other studies have shown that the transition to first birth differs by educational level (Tesching 2012). In order to compare such differences across migrant background, we did three way interactions for migrant status, education and age. However, the second generation is still quite young and so far only a small share of women with Turkish roots have both attained high education and reached ages above 30 years. Thus, the single categories in our analysis were very small and we refrain from drawing strong conclusions. Future studies about similarities and differences between age patterns of migrants’ descendants and non-migrants should be done as soon as data on at least 40 year old second generation migrants is available.

Our study adds to the literature on the fertility behaviour of migrants in advanced societies. First, in line with findings for other countries (Blau et al. 2008 for the US; Garssen and Nicolaas 2008 for the Netherlands; Parrado and Morgan 2008 for the US; Scott and Stanfors 2011 for Sweden), we were able to show a process of convergence across migrant generations in Germany. However, the second generation Turkish still differs markedly from non-migrant Germans, thus fertility adaptation seems to be less developed than for example in the Netherlands (Garssen and Nicolaas 2008). In addition, we illustrated that a distinction between the 1.5 and second generation is appropriate and necessary. From a theoretical point of view, both groups should differ in their fertility behaviour due to varying socialization experiences during childhood. In line with several migrant groups in Sweden (Scott and Stanfors 2011), our results confirm this theoretical relationship for the case of Turkish migrants in Germany. So far, only differences between 1.5 and second generation Turkish migrants concerning completed fertility have been shown (Stichnoth and Yeter 2013). We extended this to parity-specific evidence. Both the transitions to first and second childbirth have been found to differ between the two migrant generations. Furthermore, our results indicate a potential for fertility convergence in future if descendants of Turkish migrants increase their average educational attainment. Those of Turkish origin still have lower levels of education on average today, compared to non-migrant Germans. As especially the highly educated second generation has similar fertility patterns to non-migrants, the aggregated fertility of Turkish migrants should decline given an increase in educational attainment in the years to come.

For future research, in order to complete our picture of the fertility of migrants’ descendants, we should study the transition to third birth. This is of specific interest, as there might be a large difference between women in western Germany, who follow the so-called “two child norm”, and women of Turkish origin, who experience a transition to a third child more often (Milewski 2010b). In this paper, we have refrained from analysing third births which was related to the age structure of second (and partly 1.5) generation Turkish migrants in Germany who are only now reaching ages above 35 years and the number of women who are at risk of having a third birth has been still small (see Table 5 in the appendix for the number of exposures and occurrences). This will change as second generation migrants grow older. The Mikrozensus 2013 again includes the survey items on parents’ migrant status which offers the opportunity to investigate the fertility behaviour of the descendants of migrants in Germany further.

## Appendix

**Table 3:** Number of first birth events. Non-migrant Germans, 1.5 and second generation migrants. (Source: Mikrozensus 2005 and 2009, unweighted. Authors’ own calculations)

	German		1.5 generation		2nd generation	
	Share (person years)	Number of events	Share (person years)	Number of events	Share (person years)	Number of events
*Education*						
Low	18.8 %	9345	59.7 %	573	41.1 %	422
Medium	36.4 %	13,729	22.6 %	153	28.6 %	207
High	44.3 %	8775	17.7 %	50	29.6 %	73
Missing	0.5 %	153	0.5 %	4	0.7 %	3
*Age*						
18–24	11.8 %	1607	10.3 %	49	22.9 %	68
25–29	19.4 %	4682	15.6 %	119	34.5 %	253
30–34	27.5 %	9647	30.7 %	248	31.6 %	289
35–40	41.4 %	16066	43.4 %	364	11.0 %	95
*Cohort*						
1965–1972	51.0 %	19718	53.9 %	451	13.9 %	131
1973–1979	31.1 %	9391	32.1 %	253	51.3 %	416
1980–1991	17.8 %	2893	14.0 %	76	34.9 %	158
Total	100.0 %	32002	100.0 %	780	100.0 %	705

**Table 4:** Number of second birth events. Non-migrant Germans, 1.5 and second generation migrants. (Source: Mikrozensus 2005 and 2009, unweighted. Authors’ own calculations)

	German		1.5 generation		2nd generation	
	Share (person years)	Number of events	Share (person years)	Number of events	Share (person years)	Number of events
*Education*						
Low	34.8 %	5505	76.0 %	467	64.7 %	269
Medium	44.3 %	7302	17.4 %	114	25.9 %	99
High	20.3 %	4427	6.1 %	28	9.1 %	27
Missing	0.6 %	96	0.5 %	4	0.3 %	3
*Age*						
18–24	1.9 %	363	2.2 %	18	4.0 %	15
25–29	8.9 %	1848	9.8 %	80	24.7 %	116
30–34	25.6 %	5184	31.0 %	210	49.0 %	197
35–40	63.6 %	9935	57.0 %	305	22.3 %	70
*Cohort*						
1965–1972	74.0 %	11,953	67.1 %	385	30.1 %	99
1973–1979	21.7 %	4519	29.5 %	200	57.6 %	246
1980–1991	4.3 %	858	3.4 %	28	12.3 %	53
Total	100.0 %	17330	100.0 %	613	100.0 %	398

**Table 5:** Number of third birth events. Non-migrant Germans, 1.5 and second generation migrants. (Source: Mikrozensus 2005 and 2009, unweighted. Authors’ own calculations)

	German		1.5 generation		2nd generation	
	Share (person years)	Number of events	Share (person years)	Number of events	Share (person years)	Number of events
*Education*						
Low	37.0 %	1390	76.7 %	215	74.0 %	79
Medium	44.5 %	1417	19.0 %	32	21.6 %	12
High	18.5 %	805	4.3 %	4	4.4 %	3
*Age*						
18–24	0.3 %	45	0.8 %	3	1.0 %	3
25–29	4.0 %	328	7.6 %	24	18.1 %	21
30–34	21.9 %	1039	30.1 %	79	53.9 %	48
35–40	73.8 %	2212	61.4 %	147	26.9 %	23
*Cohort*						
1965–1972	80.4 %	2701	74.1 %	178	35.5 %	31
1973–1979	17.7 %	937	24.3 %	68	58.8 %	57
1980–1991	1.8 %	147	1.6 %	7	5.7 %	7
Total	100.0 %	3600	100.0 %	247	100.0 %	91

**Table 6:** Interaction migration background and education. Transition to first birth. Discrete-time hazard model (Model 2). Average marginal effects. Reference group: Second generation Turkish migrants. (Source: Mikrozensus 2005 and 2009, unweighted. Authors’ own calculations)

	2nd generation	German	1.5 generation
Low education	Ref.	− 0.046***	0.029***
Medium education	Ref.	− 0.034***	0.021**
High education	Ref.	− 0.000	0.011*

Controlled for mother’s age, cohort

*p < = 0.10; **p < = 0.05; ***p < = 0.01

**Table 7:** Descriptive statistics. Number of first birth events by migration status, education and age. (Source: Mikrozensus 2005 and 2009, unweighted. Authors’ own calculations)

Age	Education					
	Low		Medium		High	
	Share (person years)	Number of events	Share (person years)	Number of events	Share (person years)	Number of events
*German*						
18–25	71.19 %	5876	70.25 %	5985	68.35 %	1915
26–32	22.86 %	2840	24.34 %	6124	26.21 %	4895
33–40	5.94 %	346	5.41 %	843	5.44 %	1374
*1.5 migrant generation*						
18–25	82.78 %	450	81.07 %	104	66.47 %	24
26–32	14.08 %	76	16.18 %	32	26.05 %	19
33–40	3.14 %	7	2.75 %	6	7.47 %	5
*2nd migrant generation*						
18–25	85.29 %	322	85.76 %	123	77.15 %	36
26–32	13.56 %	68	13.61 %	59	20.67 %	33
33–40	1.16 %	6	0.62 %	4	2.18 %	2

**Table 8:** Three-way interaction of migration status, education and age. Transition to first birth. Discrete-time hazard model. Average marginal effects. Reference group: Second generation Turkish migrants. (Source: Mikrozensus 2005 and 2009, unweighted. Authors’ own calculations)

Age	Education		
	Low	Medium	High
*German*			
18–25	0.060	0.034	0.010
26–32	0.083	0.089	0.056
33–40	0.036	0.050	0.068
*1.5 generation Turkish*			
18–25	0.127	0.086	0.030
26–32	0.118	0.120	0.054
33–40	0.046	0.118	0.047
*2nd generation Turkish*			
18–25	0.094	0.057	0.017
26–32	0.108	0.141	0.049
33–40	0.093	0.171	0.024

*p < = 0.10; **p < = 0.05; ***p < = 0.01

**Table 9:** Interaction migration background and education. Transition to second birth. Discrete-time hazard model (Model 5). Average marginal effects. Reference group: 2nd generation Turkish migrants. (Source: Mikrozensus 2005 and 2009, unweighted. Authors’ own calculations)

	2nd generation	German	1.5 generation
Low education	Ref.	− 0.048***	0.038**
Medium education	Ref.	− 0.003	0.093**
High education	Ref.	0.078**	0.047

Controlled for years since first birth, mother’s age at first birth and birth cohort

*p < = 0.10; **p < = 0.05; ***p < = 0.01

## Footnotes

1 It should be noted that the definition of the 1.5 generation migrants differ across studies. Seibert (2008) defines 1.5 generation migrants as those who arrived in Germany before age 15. Segeritz et al. (2010) and Fick (2011) refer to those who arrived in Germany by school starting age (6 years).

2 In order to clearly distinguish between second and third generation migrants, we would need information not only on parents’ citizenship but on their place of birth, which is not available for all respondents in the Mikrozensus. However, we argue that third generation Turkish migrants are only reaching adulthood now and thus are only to a minor extent considered in the age groups under study.

3 Results available upon request.

4 Results available upon request.
